# The role of Traditional Chinese medicine in anti-HBV: background, progress, and challenges

**DOI:** 10.1186/s13020-023-00861-2

**Published:** 2023-12-02

**Authors:** Feilin Ge, Yan Yang, Zhaofang Bai, Lanlan Si, Xuemei Wang, Jia Yu, Xiaohe Xiao, Yan Liu, Zhigang Ren

**Affiliations:** 1 Department of Chinese Medicine, The First Affiliated Hospital of Zhengzhou University, Zhengzhou, 450052 China; 2 College of Traditional Chinese Medicine, Chongqing Medical University, Chongqing, 400010 China; 3 The Fifth Medical Center, Chinese PLA General Hospital, Beijing, 100039 China; 4 Department of Infectious Diseases, The First Affiliated Hospital of Zhengzhou University, Zhengzhou, 450052 China

**Keywords:** Traditional Chinese medicine, Hepatitis B virus, Nucleos(t)ide analogues, Functional cure, Antiviral mechanism

## Abstract

Chronic hepatitis B (CHB) remains a major world's most serious public health issues. Despite the remarkable effect of nucleos(t)ide analogues (NAs) in inhibiting hepatitis B virus (HBV) deoxyribonucleic acid (DNA) as the first-line drug, there are several limitations still, such as poor antigen inhibition, drug resistance, low-level viremia, restricting patients' functional cure. Due to the constraints of NAs, traditional medicines, such as traditional Chinese medicine (TCM), have become more prevalently used and researched in the clinical treatment of CHB as complementary alternative therapies. As a consequence, the review focuses on the background based on HBV’s life cycle as well as the NAs’ limitations, progress based on direct and indirect pathway of targeting HBV of TCM, and challenges of TCM. We found TCMs play an increasingly important role in anti-HBV. In a direct antiviral way, they regulate HBV infection, replication, assembly, and other aspects of the HBV life cycle. As for indirect way, TCMs can exert anti-HBV effects through targeting the host, including immune regulation, apoptosis, autophagy, oxidative stress, etc. Especially, TCMs have the advantages of strong antigenic inhibition compared to NAs. Specifically, we can combine the benefits of TCMs in strong HBV antigen inhibition with the benefits of NAs in targeted antiviral effects, in order to find a suitable combination of "TCM + NAs" to contribute to Chinese knowledge of the realisation of the “global elimination of HBV by 2030” goal of the World Health Organization.

Infection with the hepatitis B virus (HBV) causes chronic hepatitis B (CHB) and raises the risk of cirrhosis and hepatocellular carcinoma (HCC), making it an extremely infectious disease that endangers human health. Studies have reported that there are approximately 257 million patients worldwide with positive hepatitis B surface antigen (HBsAg), resulting in more than 887,000 deaths per year [1, 2]. CHB is the leading cause of liver cancer, and about 92.05% of liver cancer is associated with CHB [3]. Even though the effective application of HBV vaccine has greatly reduced the number of new cases, according to the statistics from 2010 to 2022, the number of new cases of CHB is still 900,000 to 1 million per year in China alone [4]. Therefore, CHB remains one of the world's most serious public health issues. Current clinical anti-HBV drugs are primarily nucleos(t)ide analogues (NAs) that inhibit HBV polymutase/reverse transcriptase activity and the immunomodulator interferon (IFN), with NAs being more widely used because of their oral convenience, rapid onset of action, and relatively low side effects. There are two IFNs (pegylated interferon (PegIFN), interferon-alfa (IFN-α)) are approved for clinic [2]. There are four NA (lamivudine (LAM), entecavir (ETV), telbivudine (LdT) and emtricitabine (FTC)) and three NAs (adefovir dipivoxil (ADV), tenofovir disoproxil fumarate (TDF) and tenofovir alafenamide (TAF)) are approved for clinic [2]. Functional cure, defined as HBV DNA-negative and HBV antigen-negative, is the ideal treatment outcome for CHB patients. However, the deficiencies of NAs, such as poor antigen inhibition and drug resistance, restrict the functional cure of CHB patients and severely limit the World Health Organization's (WHO) goal of global elimination of HBV by 2030 [5, 6].

Given the constraints of currently available anti-HBV drugs, traditional medicines such as traditional Chinese medicine (TCM) have been progressively utilized as complementary alternative therapies in the clinic. TCM has multi-constituent, multi-pathway, and multi-target characteristics. The ideal antiviral drug is a combination treatment that targets multiple stages of the viral life cycle, such as virus entry, decapitation into the nucleus, transcriptional replication, and assembly, enabling the virus to be removed from the root. Fortunately, the characteristics of TCM correspond to the goals of ideal antiviral drugs. Therefore, TCM may have prospective value and advantages in the treatment of HBV, notably in bridging the NAs’ deficiency and promoting functional cure. Studies have shown that TCMs such as *Phyllanthus amarus* Schumach. & Thonn., *Scutellaria baicalensis* Georgi., *Sophora flavescens* Aiton,. *Curcuma longa* L. and*Rheum palmatum* L. have shown better anti-HBV effects and clinical application prospects, with some having the capacity of promoting functional cure [7–9]. In summary, the review focuses on the background, progress, and challenges of TCM and their active constituents in anti-HBV based on the HBV life cycle, as well as the limits of NAs.

## The life cycle of HBV

### HBV infects the host liver cells and uncoatings in the nucleus

An early phase in the HBV replication cycle is HBV entrance into host liver cells and uncoating in the nucleus. When HBV viral particles (Dane particles) approach the hepatocyte surface, the small hepatitis B surface antigens (SHBs) and large hepatitis B surface antigens (LHBs) on their envelope bind to heparan sulfate proteoglycan (HSPG) and sodium taurocholate cotransporting polypeptide (NTCP) on the hepatocyte membrane respectively, and facilitate viral particle entrance into hepatocytes via clathrin-dependent endocytosis into hepatocytes. HBV enters the hepatocyte by endosomal escape, exposing the nucleocapsid, which then transports along the intracytoplasmic microtubules to the nucleus, where it separates from the microtubules and binds to importin α/β. The nuclear capsid then passes through the nuclear pore complex, dissociates from importin α/β, and binds to Nup153. Eventually, the mature nuclear capsid dissolves into free HBcAg, allowing relaxed-circular DNA (rcDNA) to enter the nucleus [10]. The rcDNA that enters the nucleus of the host liver cells will be converted into cccDNA.

### Transcription, translation and proliferation of cccDNA

**Fig. 1:**
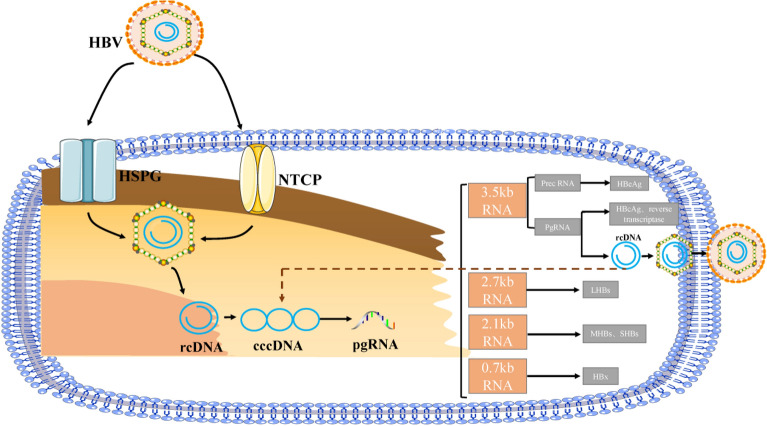
The life cycle of HBV

The template for HBV replication and the source of persistent HBV infection is cccDNA, which exists in the nucleus of host hepatocytes. Through transcription and translation, rcDNA is repaired to cccDNA for HBV replication and proliferation. cccDNA can be transcribed into 5 different RNA fragments of 3.5 kb (2 RNAs), 2.4 kb, 2.1 kb, and 0.7 kb, which can then be translated into 7 different proteins. Specifically, 3.5 kb RNA contains two types of RNA: Prec RNA and pg RNA. Prec RNA translates into HBeAg, whereas pgRNA not only translates into HBcAg and reverse transcriptase, and reverse transcribes into rcDNA also; 2.4 kb RNA translates into LHBs. 2.1 kb RNA translates into SHBs and middle hepatitis B surface antigens (MHBs); 0.7 kb RNA translates into HBx. pgRNA is reverse transcribed into rcDNA, which can then be assembled into new HBV viral particles by HBsAg, HBcAg, HBx, and other viral proteins and secreted extracellularly to infect other liver cells. rcDNA, on the other hand, can generate cccDNA by repairing both the positive and negative strands to form a new HBV replication template. Notably, cccDNA and rcDNA can integrate into host DNA, which is a main cause of HBV persistence as well as HCC [11] (Fig. 1).

## Current limitations of first-line anti-HBV drugs

NAs and IFNs are the first-line anti-HBV drugs recommended by clinical guidelines, with NAs being more widely used owing to the ease of oral administration, rapid efficacy, and relatively low side effects. Therefore, the following focuses on the limitations of NAs.

### Current limitations of NAs

**Fig. 2:**
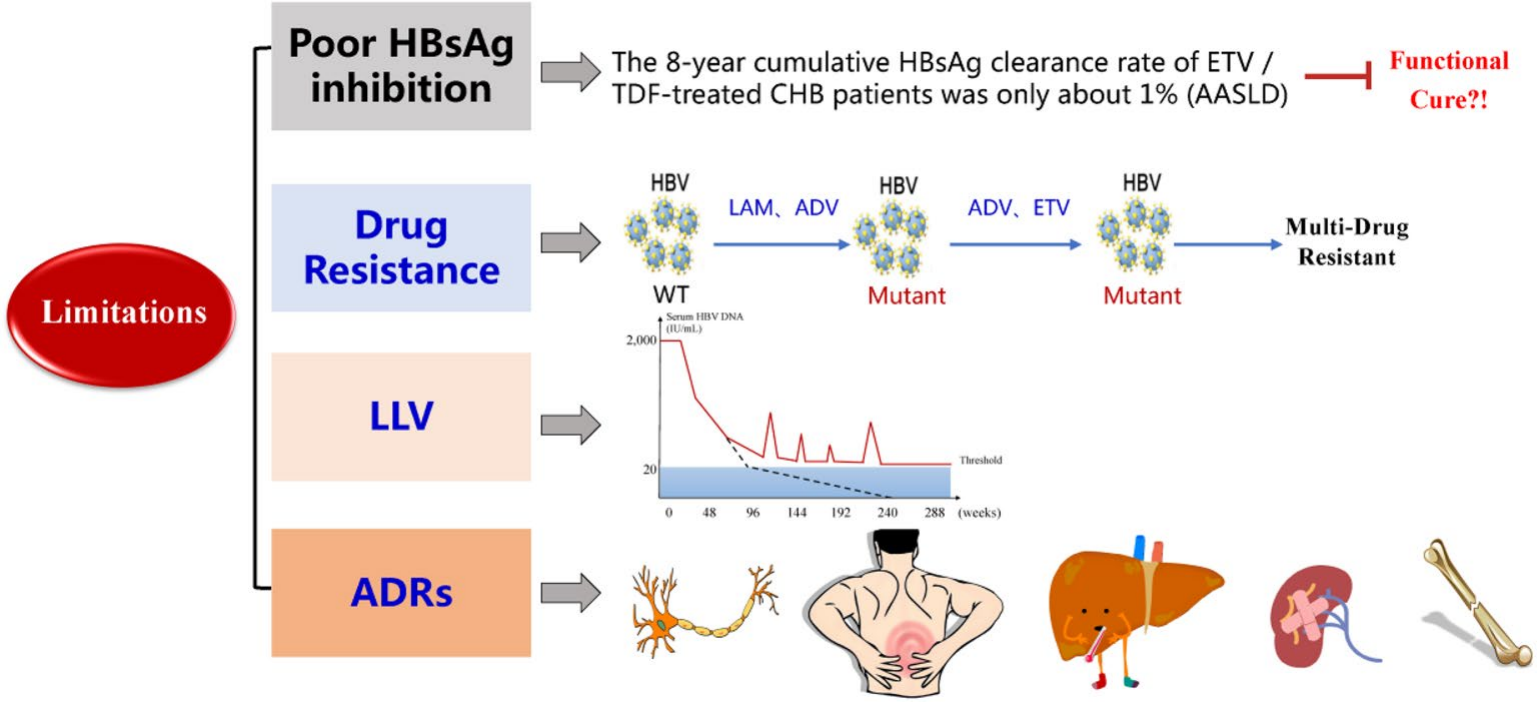
Current limitations of NAs

Although NAs are effective in suppressing HBV DNA and have demonstrated positive clinical efficacy, they still have many limitations that make achieving a functional cure in CHB patients difficult [2–4]. The limitations of NAs will be discussed in the subsequent, primarily in terms of poor HBsAg inhibition, drug resistance, low-level viremia (LLV), and adverse drug reactions (ADRs), in order to serve as a reference for the development of new drugs for HBV functional cure (Fig. 2).

#### Poor HBsAg inhibition

While it is true that NAs have such a significant inhibitory effect on HBV DNA, their inhibition/clearance of HBsAg is minimal. A real-world cohort study, presented in November 2021 at the annual meeting of the American Liver Association, revealed that the 8-year cumulative HBsAg clearance rate of ETV/TDF-treated CHB patients was only about 1% [12]. The biological function of HBsAg is closely related to its role as a crucial indicator of HBV functional cure [13]: first, HBV enters liver cells by binding to HSPG and NTCP on hepatocyte membranes via HBsAg. Second, HBsAg can decrease the body's immune response by depleting T cells. Under normal conditions, HBsAg binds to human leukocyte antigen I (HLA-I) in hepatocytes as a complex and becomes a T-cell epitope, while cytotoxic T lymphocytes (CTL) clear HBV by recognizing the epitope (HBsAg/HLA-I complex). HBsAg does provide immune clearance of HBV in the early stages by activating T lymphocytes. However, HBV is very "shrewd" in that it activates T lymphocytes by secreting a large number of non-infectious sub viral particles (SVPs) containing only HBsAg empty shells, resulting in immune clearance of "fake" HBV. Long-term high SVP concentrations may result in CTL cell failure [14]. Moreover, HBsAg induces immune tolerance by suppressing the immune response, enabling HBV to escape immune detection. In summary, the biological function of HBsAg determines that HBsAg is the primary cause of persistent HBV infection and even HCC [15–26], and NAs' pretty narrow HBsAg inhibition/clearing effect severely restricts functional cure.

#### Drug resistance

NAs resistance commences with genotypic resistance, then progresses to virological and biochemical breakthrough, and ultimately clinical resistance, with resistance being closely related to its antiviral mechanism [27–31]. Our team's analysis of 28,236 patients treated with NAs who received HBV resistance testing at the Fifth Medical Center of the Chinese People's Liberation Army General Hospital over the last 12 years (2008–2019) disclosed that clinical NAs drug use was complex and diverse, and that, whereas the guideline-recommended dosage of non-first-line NAs decreased and first-line drugs increased, the composition ratio of multidrug-resistant HBV mutation detection increased year by year, with the overall detection rate reaching 6.56%. TDF and TAF are effective in the treatment of drug-resistant HBV infection, but they still belong to NAs that directly target the reverse transcriptase of HBV, and when they are used to treat patients with multidrug-resistant HBV infection, close to 30% patients still have poor virological responses or virological breakthroughs during treatment [32–34]. HBV has been reported in recent years to develop quadruple resistance mutations against TDF, which tends to result in treatment failure [35]. Once drug resistance develops, the liver disease process accelerates, causing the emergence of liver fibrosis, cirrhosis, and HCC, which endangers CHB infection patients [36, 37]. Improving the efficacy of patients with multidrug-resistant HBV infection is thus a vital issue.

HBV mutation and drug resistance are the result of selective evolution of the HBV under the continuous pressure of antiviral drugs. The CHB treatment strategy that blindly uses direct suppression of HBV as the main means, which may become more and more difficult to achieve the therapeutic purpose due to virus mutation and drug resistance [38]. In fact, HBV infection is a dynamic process of the interaction of virus, liver cells and the body's immune system. CHB is not only a viral infectious disease, but also a body immune disease. The immune system plays an important role in HBV infection and treatment. To focus on the body factors, the traditional Chinese medicine "Fuzheng Quxie" treatment principle is adopted. On the basis of antiviral drug treatment, combined with “kidney nourishing liver detoxification” Chinese medicine, hoping to regulate the immune response level of the body to increase the immune system's clearance ability to HBV, so as to reduce the occurrence of HBV resistance and improve the prognosis of drug-resistant HBV [39].

#### LLV

There is no accepted definition of LLV. Based on the 2018 American Liver Association guidelines [40], patients with detectable HBV DNA of < 2000 IU/ml (with a minimum detection limit of 10 IU/ml) have LLV. LLV is defined by Chinese scholars as serum HBV DNA < 2000 IU/ml in CHB patients treated with first-line NAs for at least 48 weeks after excluding adherence problems and viral resistance mutations, while still detectable by quantitative real-time polymerase chain reaction (qRT-PCR) [28, 41–44]. LLV has a negative impact on the prognosis of CHB patients, primarily by reducing reversal rate of liver fibrosis and significantly increasing the risk of HCC in cirrhotic patients. A clinical study identified that after 78 weeks of treatment, patients with progressive liver fibrosis had a 50% HBV DNA detection rate, which was significantly higher than patients with reversal of liver fibrosis (19%) [45]. Another cohort study, using a more sensitive assay, discovered that low positive values of HBV DNA and pgRNA under NAs treatment were linked to the development of HCC [46–49]. Despite the lack of high-quality clinical evidence regarding the poor prognosis of LLV, patients with LLV must be closely monitored.

#### ADRs

There are significant challenges in the safety of NAs, which are associated with renal, muscular, skeletal, and neurological ADRs in anti-HBV therapy, with renal and skeletal predominating. Renal impairment occurs in 2.6% and 3–12% of CHB patients treated with TDF and ADV, respectively [50, 51]. In terms of skeletal injury, both were able to significantly increase the risk of hip fracture [52, 53]. Muscle injury was the most common ADR of LAM and LdT, with incidences of 9–12.9% and 4.1%, respectively [54]. Peripheral neuropathy appeared in approximately 1.9–7.3% of CHB patients treated with LdT [55]. Data mining of ADRs using the Food and Drug Administration (FDA)'s Adverse Event Reporting System (FAERS) database [56–58] revealed that ETV ADRs mainly included systemic damage, hepatobiliary damage, and urinary damage, whereas TDF primarily included urinary damage, skeletal-muscular damage, metabolic and nutritional disorders, and so on.

### Current limitations of IFNs

IFNs are not recommended for patients with decompensated liver cirrhosis, liver transplantation, severe acute HBV infection, pregnancy, severe depression [1]. IFNs have many ADRs, such as flu-like symptoms, bone marrow suppression, and semen abnormal symptoms, but most ADRs are temporary and symptomatic Deal. In addition, IFNs are more expensive, some patients may not be able to afford it for long [2].

## Progress of TCM and its constituents against HBV

### Overview of TCM for the treatment of CHB

Given the drawbacks of existing anti-HBV drugs, TCM has been increasingly used in the clinic as a complementary and alternative therapy. TCM has been applied to treat more than 90% Chinese CHB patients, and more than 80% of TCM is considered effective in the treatment of liver disease [5]. By referencing the published research reports related to anti-HBV, we reviewed the most common TCMs and their constituents. The TCM compound with anti-HBV effects include Su-duxing (SDX), Liuweiwuling tablets (LWWL), Yinchenhao decoction, Xiaochaihu decoction, and Yexiazhu, etc. The TCMs include *Artemisia scoparza* Waldst.et Kit., *Artemisia annua* L., *Salvia miltiorrhiza* Bge., *Polygonum cuspidatum* Sieb.et Zucc., *Sophora flavescens* Aiton, etc. The constituents include quercetin, luteolin, wogonin, oxymatrine, sophoridine, aloe emodin, danshensu, rosmarinic acid, lithospermic acid, resveratrol, and Saikosaponin C, etc. [59–62].

### Research progress of TCM in anti-HBV

The mechanism of TCM against HBV mainly involves two aspects: direct pathway of targeting the HBV and indirect pathway of targeting the host, with the latter being the primary. The direct pathway mainly targets various stages of the HBV life cycle, such as inhibition of HBV invasion, targeting HBV cccDNA and inhibition of HBV transcription. Indirect pathways include immune modulation, apoptosis, autophagy, oxidative stress and others, among which the immune modulation being the most frequently reported.

#### Direct pathway of targeting HBV

##### Inhibition of HBV invasion into host liver cells

HBV initiates its life cycle by entering intracellularly via NTCP and clathrin on the surface of host liver cells. Therefore, by inhibiting NTCP and clathrin, TCMs have the potential to inhibit HBV invasion, infection and replication at the source [63, 64]. Tsukuda reported that proanthocyanidin inhibited HBV entry into host hepatocytes by inhibiting the binding of HBsAg to NTCP, and it inhibited HBV of different genotypes [65]. Umetsu et al. discovered that silibinin inhibited HBV invasion by inhibiting clathrin-mediated endocytosis and decreasing transferrin uptake [66]. Huang et al. unearthed that epigallocatechin-3-gallate induced NTCP transfer from the cytosol to the cytoplasm as well as promoted the degradation of NTCP by lysosome, thereby inhibiting HBV invasion into hepatocytes [67].

##### Targeted inhibition of HBV cccDNA

Since the persistence and stability of HBV cccDNA is a key element in the difficulty of functional cure in CHB patients, targeted inhibition/silencing of cccDNA has always been a hot and tough area of anti-HBV [68–70]. Bai et al. [71] demonstrated that luteolin, saikosaponin C, and baicalin inhibited HNF-4α expression by activating ERK signaling. Punicalagin and three other tannic acids were revealed to inhibit cccDNA formation, promote cccDNA degradation, and reduce cccDNA levels by Liu et al. [72]. Curcumin, according to Wei et al. [73], can disrupt cccDNA homeostasis by lessening the acetylation levels of cccDNA histones H3 and H4.

#### Indirect pathway of targeting the host

##### Modulation of immune response to anti-HBV

The host's struggle against HBV is predominantly a constant battle between the host immune system ("self") and HBV ("enemy"). The body is in equilibrium when the host immune system is evenly matched with HBV, the host is only a carrier of HBV. When the host immune system is stronger than HBV, the "self" defeats the "enemy", and the immune system will clear a large amount of HBV, which may be accompanied by liver inflammation damage. However, when HBV is stronger than the immune system, the "enemy" will overpower the "self", and the host will be in an immune tolerance state, which may eventually lead to liver inflammation damage, which can result in end-stage liver diseases like cirrhosis and HCC [2, 5]. As a result, enhancing patients' immune capacities is critical for HBV clearance/suppression. Many TCMs are natural immune enhancers and can exert antiviral effects by boosting immune capacity.

Specifically, TCMs' modulation of immune response to anti-HBV includes the regulation of innate immunity along with adaptive immunity. To exert anti-HBV effects, TCM can activate innate immune signaling pathways such as TLR7/TLR9, RIG-I, and c-GAS to promote the production of IFN. In addition, it can promote the expression of cytokines such as IL-1β, IL-6, IL-12, and TNF-α to improve the immune clearance of HBV. In previous studies [6, 74], we noticed that the LWWL and SDX had significant inhibitory effects on HBV DNA, HBsAg, and pgRNA levels both in vitro and in vivo, particularly in inhibiting HBsAg and HBcAg by more than 50%, and also had significant inhibitory effects on drug-resistant HBV, with obvious advantages of comprehensive anti-HBV effects. Further transcriptomic analysis and experimental validation confirmed the effect was tightly connected to RIG-I/IFN-β pathway activation. Yao et al. disclosed that oxymatrine can stimulate the TLR9/IFN-α pathway and promote the production of IFN-α, an antiviral effector molecule [75]. According to Fang et al. [76], lentinan can increase the expression of cytokines including IL-1β, IL-12, and TNF-α in monocytes/macrophages, antagonizing HBsAg-mediated immune tolerance, and the mechanism may be related to the activation of the MAPK and NF-κB pathways.

With regard to the regulation of adaptive immunity by TCMs, they exert anti-HBV effects by activating CD4^+^ T cells to promote IFN-γ and by regulating different T cell subsets such as Th1 and Th17. Heretofore, we found that LWWL and oxymatrine could activate CD4^+^ T cells to promote INF-γ, and that the SDX could activate different T cell subpopulations [6, 8, 74]. Liu et al. [77] reported that nourishing kidney and eliminating toxicity decoction could inhibit Th17 cells and activating Th1 cells, thereby enhancing immune clearance of HBV. It is worth noting that in adaptive immunity, cells such as CTL cells and B cells are also significant elements for HBV clearance, however, no relevant studies on anti-HBV by activation of CTL cells and B cells by TCM have been reported.

##### Apoptosis of HBV-infected hepatocytes

In addition to immune clearance of HBV, TCMs may also exert antiviral effects by inducing apoptosis in HBV-infected hepatocytes. Nevertheless, the mechanism of HBV infect is complex and unclear, in which apoptosis related is obscure still. A few studies suggest that HBV can trigger hepatocyte apoptosis and induce damage, while the majority of studies suggest that HBV can suppress apoptosis [78–80]. We contend that HBV inhibits the host's hepatocytes apoptosis. It is known that HBV does not cause harm, and liver damage is primarily caused by inflammatory damage induced by an HBV-mediated immunological response. Therefore, HBV may increase the microenvironment for its survival by inhibiting the apoptosis of host cells. Our pre experimental results corroborated this hypothesis, since the apoptotic effect was dramatically reduced in the liver of HBV model mice compared to control. In summary, various research have investigated the antiviral mechanism of TCMs from the perspective of regulating apoptosis-related pathways to inhibit HBV, based on the virulence mechanism that HBV promotes its survival by inhibiting the apoptotic effect. Polysaccharides extracted from sipunculus nudus and humic acid, for example, were found to influence the CASP3 pathway, while *Artemisia argyi Levl*. et vant was found to regulate the Wnt/β-catenin pathway, triggering dose-dependent apoptosis in HepG2 2.15 cells and limiting HBV DNA replication [81–83].

##### Inhibition of autophagy in HBV-infected hepatocytes

HBV can accelerate self-replication in the early stages of infection by eliciting inadequate autophagy in host cells. As a result, TCMs can impede this connection to anti-HBV. Pant et al. observed that one of the major constituents of mineral pitch, humic acid, can limit viral replication by decreasing HBx expression and causing a decrease in autophagosomes of HBV-infected hepatocytes [84]. Zhong et al. discovered that epigallocatechin-3-gallate could limit viral multiplication by increasing lysosomal acidity and decreasing autophagosomes in HBV-infected liver cells. Notably, decreasing cellular autophagy in the early stages of HBV infection can have antiviral effects, however, after progression to HCC is no longer recommended since autophagy can have anti-tumor effects by promoting tumor cell death [85, 86].

##### Inhibition of oxidative stress in HBV-infected hepatocytes

**Fig. 3:**
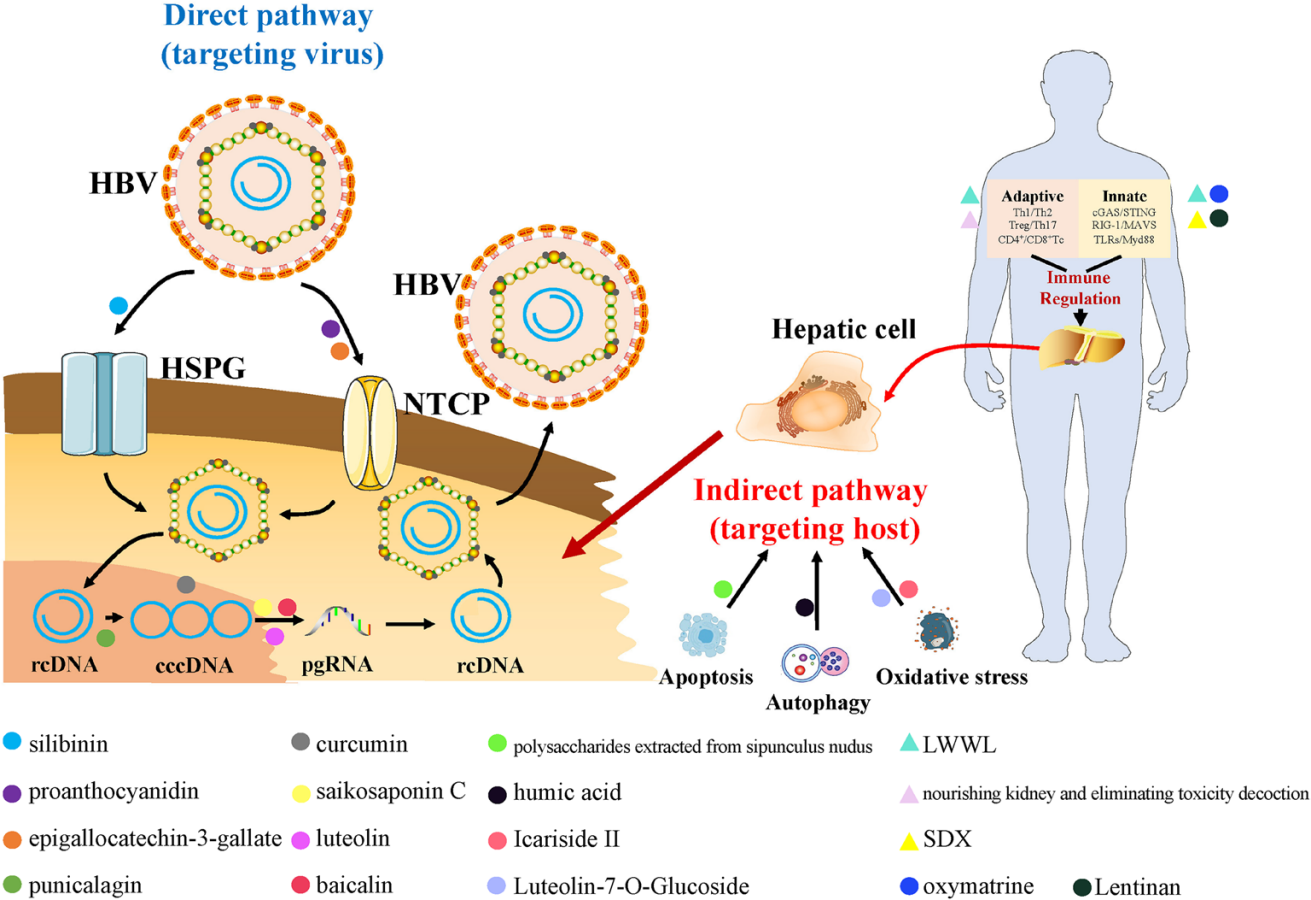
Pathway of TCM in anti-HBV

HBV viral proteins accumulate for a long time in the endoplasmic reticulum of host hepatocytes, exacerbating endoplasmic reticulum stress and oxidative DNA damage [87, 88]. As a result, anti-HBV medicines can have an indirect antiviral effect via oxidative stress inhibition. Arbab et al. revealed that the extract of *Guiera senegalensis* J.F. Gmel. could protect hepatocytes from HBV-induced oxidative stress [89]. Cui et al. proved that luteolin-7-*O*-Glucoside could improve mitochondrial function by inhibiting ROS and, as a result, inhibit HBV antigen secretion. In vitro and in vivo studies imply that icariside II's anti-HBV effect is closely related to the regulation of GSH, GST, and SOD levels to inhibit oxidative stress [90] (Fig. 3; Table 1).

**Table 1:** Mechanism of TCM in anti-HBV

Term	Anti-HBV mechanism
Liuweiwuling tablets^#^	Stimulating the production of IFN-γ and IFN-β to activate CD4+ T cells
Nourishing kidney and eliminating toxicity decoction^#^	Enhancing the immune system's ability to clear HBV by activating Th1 cells and suppressing Th17 cells
Su-duxing^#^	Activating various T cell subtypes to combat viral infections
Silibinin^*^	Inhibiting HSPG to suppress HBV cell entry
Proanthocyanidin^*^	Inhibiting the binding of HBsAg to NTCP to suppress HBV entry into liver cells
Epigallocatechin-3-gallate^*^	Inducing the translocation of NTCP from the cell membrane to the cytoplasm while promoting lysosomal degradation of NTCP
Punicalagin^*^	Reducing the cccDNA (covalently closed circular DNA) levels
Curcumin^*^	Disrupting cccDNA equilibrium
Saikosaponin C^*^	Activating the ERK signal transduction pathway to disrupt HBV transcription and replication
Luteolin^*^	Activating the ERK signal transduction pathway to disrupt HBV transcription and replication
Baicalin^*^	Activating the ERK signal transduction pathway to disrupt HBV transcription and replication
Polysaccharides extracted from sipunculus nudus^*^	Modulating the Wnt/β-catenin pathway to induce apoptosis
Humic acid^*^	Inhibiting HBx expression to reduce autophagosomes induced by HBV infection in liver cells, thereby suppressing viral replication
Icariside II^*^	Regulating the levels of GSH, GST, and SOD to suppress oxidative stress
Luteolin-7-*O*-Glucoside^*^	Inhibiting ROS (Reactive Oxygen Species)
Oxymatrine^*^	Activating the TLR9/IFN-α signaling pathway to promote IFN-α production
Lentinan^*^	Activating the MAPK and NF-κB signaling pathways

^#^TCM compound; ^*^TCM constituents

## Privileges and challenges of TCM in anti-HBV

### Privileges

TCMs offer unique advantages in anti-HBV. (1) TCMs have the characteristics of being multi-constituent, multi-target, and multi-pathway, allowing them to integrate different pathways to exert antiviral effects [59–62]. For one thing, TCMs have the capability to regulate multiple immune cells/pathways/factors, including innate and adaptive immunity, and exert immune clearance effects. For another, it has the ability to act directly on numerous phases of the HBV life cycle, including virus infection, replication, and assembly, to exert direct antiviral effects. Take LWWL as an example, in terms of anti-HBV constituents, LWWL contains quercetin, luteolin, wogonin, and others, which is the characteristic of multi-constituent. In terms of mechanism, LWWL may be closely related to its promotion of immunity, which primarily involves the promotion of IFN-γ and IFN-β production (innate immunity), activation of CD4^+^ T cells (adaptive immunity). Besides, TCM is multi-target and multi-pathway, it may specifically induce apoptosis in HBV-infected cells [74]. (2) TCMs are not associated with viral drug resistance [6, 8, 74]. Because of the multi-target, it is not easy for TCMs to develop drug resistance against a specific target, and there have been no reports of drug resistance. SDX, a TCM compound, can inhibit both wild-type and ETV-resistant HBV, and has no HBV resistance [6]. (3) It is well comprehended that HBV antigen clearance is essential for functional HBV cure, which is the biggest deficiency in the efficacy of current NAs. TCMs are far more effective than NAs in inhibiting HBV antigens (particularly HBsAg) and have the potential of functional cure [91, 92]. According to studies, both LWWL and SDX achieve more than 50% HBsAg inhibition [6, 74], which is superior to NAs. (4) Furthermore, there are few ADRs associated with TCMs [5], which are always their advantages as natural drugs, and there was no statistical difference in the incidence of ADRs in the combination group compared with NAs alone group in clinical studies.

### Challenges

Despite TCMs’ distinct privileges in anti-HBV, there are significant challenges. (1) The anti-HBV effect of TCM is not clear, which seriously restricts the improvement of anti-HBV effect. For example, the TCM compounds SDX and LWWL mentioned in this paper, although they have shown good effects against HBV in vivo and vitro, especially in the HBV antigen inhibition, effective components are uncertain, which limits clinical application. (2) The anti-HBV mechanism of most TCM is general and not deep enough. The direct action target and interaction mechanism of TCM against HBV are still unclear, which is the bottleneck restricting the advantages of TCM against HBV. (3) Since TCM is always used as a complementary therapy in combination with NAs in the clinic, evaluating the antiviral effect of TCM alone is difficult. (4) Due to the large number of constituents in TCM, standardization is tricky, resulting in large differences in the efficacy of the same TCM from different origins/manufacturers. (5) Compared with NAs, TCM has a weaker role in direct antiviral targeting of HBV.

## Overview/Summary

Although NAs are effective in clinically treating CHB patients by inhibiting HBV DNA, there are still many limitations, such as poor HBV antigen inhibition, drug resistance, LLV, and more ADRs, limiting the functional cure. TCMs play an increasingly important role in anti-HBV. In a direct antiviral way, they regulate HBV infection, replication, assembly, and other aspects of the HBV life cycle. As for indirect way, TCMs can exert anti-HBV effects through targeting the host, including immune regulation, apoptosis, autophagy, oxidative stress, etc. Especially, TCMs have the advantages of strong antigenic inhibition, no drug resistance, and fewer ADRs when compared to NAs. As a consequence, we should fully dig the benefits in anti-HBV. We can combine the benefits of TCMs in strong HBV antigen inhibition and no drug resistance with the benefits of NAs in targeted antiviral effects, particularly HBV DNA inhibition, and actively seek a "TCM + NAs" combination that maximizes drug efficacy to compensate the shortcomings of NAs. We aspire to contribute to Chinese knowledge of the realisation of "global HBV elimination by 2030".

## Data Availability

No data was used for the research described in the article.
